# MRI-based assessment of liver perfusion and hepatocyte injury in the murine model of acute hepatitis

**DOI:** 10.1007/s10334-016-0563-2

**Published:** 2016-05-09

**Authors:** Katarzyna Byk, Krzysztof Jasinski, Zaneta Bartel, Agnieszka Jasztal, Barbara Sitek, Boguslaw Tomanek, Stefan Chlopicki, Tomasz Skorka

**Affiliations:** 1 Department of Magnetic Resonance Imaging, Institute of Nuclear Physics, Polish Academy of Sciences, Krakow, Poland; 2 Jagiellonian Centre for Experimental Therapeutics (JCET), Jagiellonian University, Krakow, Poland; 3 Division of Medical Physics, Department of Oncology, University of Alberta, Edmonton, Alberta Canada; 4 Department of Experimental Pharmacology, Chair of Pharmacology, Medical College, Jagiellonian University, Krakow, Poland

**Keywords:** Acute liver failure, Arterial spin labelling, DCE-MRI, Empirical mathematical modelling, Perfusion

## Abstract

**Objective:**

To assess alterations in perfusion and liver function in the concanavalin A (ConA)-induced mouse model of acute liver failure (ALF) using two magnetic resonance imaging (MRI)-based methods: dynamic contrast-enhanced MRI (DCE-MRI) with Gd-EOB-DTPA contrast agent and arterial spin labelling (ASL).

**Materials and methods:**

BALB/c mice were studied using a 9.4 T MRI system. The IntraGateFLASH^TM^ and FAIR-EPI pulse sequences were used for optimum mouse abdomen imaging.

**Results:**

The average perfusion values for the liver of the control and ConA group were equal to 245 ± 20 and 200 ± 32 ml/min/100 g (*p* = 0.008, respectively). DCE-MRI showed that the time to the peak of the image enhancement was 6.14 ± 1.07 min and 9.72 ± 1.69 min in the control and ConA group (*p* < 0.001, respectively), while the rate of the contrast wash-out in the control and ConA group was 0.037 ± 0.008 and 0.021 ± 0.008 min^−1^ (*p* = 0.004, respectively). These results were consistent with hepatocyte injury in the ConA-treated mice as confirmed by histopathological staining.

**Conclusions:**

Both the ASL and DCE-MRI techniques represent a reliable methodology to assess alterations in liver perfusion and hepatocyte integrity in murine hepatitis.

## Introduction

Acute liver failure is a disease associated with high mortality and multiorgan dysfunction [1]. The aetiology of ALF is complex [2] as it can be caused by viruses, reactions to drugs, vascular diseases or metabolic syndromes [2, 3]. ALF is associated with a primary liver dysfunction, activation of pro-inflammatory responses including pro-inflammatory cytokines, acute phase proteins [4], and activation of liver sinusoidal endothelial cells (LSEC), Kupffer cells and recruitment of immune cells [5]. ALF invariably leads to the loss of hepatocyte integrity as mirrored by the elevated levels of the liver specific enzymes [alanine aminotransferase (ALT) and aspartate aminotransferase (AST)] in blood [6]. It also influences the functional state of the liver leading to haemodynamic disturbances, inappropriate vasodilatation and reduction of perfusion [7–9].

The ConA-induced model of ALF was originally used for studies of the pathophysiology of lymphocyte T-dependent liver injury and in pre-clinical studies on liver inflammation [10]. ConA–induced hepatitis involves cooperative activation of natural killer T (NKT) with conventional T cells and Kupffer cells and IL-4-, TNF-α- and IFN-γ-mediated liver inflammation in mice with subsequent development of hepatocellular apoptosis and necrosis [10–12] that eventually results in endothelium disintegration and necrosis of hepatocytes [10].

MRI-based methods can be applied for the quantitative measurement of liver function in hepatitis using small animals models; however the studies are challenging because of the low signal-to-noise ratio and respiratory artefacts. Animals with advanced ALF may develop complications such as acute lung injury or respiratory distress syndrome [1] causing uncontrolled motion and thus further image distortions. Therefore proper pulse sequences must be selected to ensure optimum image quality.

Complex diagnosis of ALF includes investigation of hepatocyte integrity and liver perfusion. The MRI technique, arterial spin labelling (ASL), was developed for perfusion measurements [13]. The double-triggered flow-sensitive alternating inversion recovery (FAIR) [14] with rapid single-shot echo planar imaging (EPI) acquisition is a particular type of ASL. FAIR registers blood inflows from different directions [14]; therefore it is advantageous over other types of ASL modalities for perfusion imaging of the liver with a complex microcirculatory bed. Moreover, the FAIR-EPI technique allows obtaining maps of the *T*_1_ relaxation time, which was previously reported as an indicator of liver disease [15, 16]. DCE-MRI was used in studies of liver injury [17, 18], but was also reported to be useful for assessment of perfusion changes [19, 20]. The *T*_1_-weighted fast gradient echo sequences are used for DCE-MRI, but they need triggering. Therefore the self-gated IntraGateFLASH^TM^ [21] technique seems valuable especially for small animal imaging. This sequence allows continuous scanning in steady-state conditions and retrospective removal of the data altered by respiration artifacts. IntraGateFLASH^TM^ has been used in studies of mouse heart mapping [22] and mouse cholangiography [23], for example.

The aim of our work was to measure perfusion and hepatocyte injury in the mouse model of acute hepatitis induced with ConA with the use of two complementary MRI-based methods. Alterations in the hepatocyte integrity and functional state of microcirculation of the liver were assessed as they are important factors determining liver function and response to injury.

## Materials and methods

### Animals studies

Fourteen 3-month-old BALB/c mice were used in this study. Acute hepatitis was induced by ConA (Sigma-Aldrich, USA) administered intravenously. MRI measurements were performed 24 h after ConA injection. The animals were divided into the control and ConA group (*n* = 7 in each group). The treated group received 8 mg/kg b.w. ConA in 1 M phosphate-buffered saline (PBS), and the control group received the same volume of saline. The mice were fed a standard laboratory diet and were maintained in a 12-h light/dark cycle at 23 °C temperature. All procedures were approved by the local Ethics Committee on Animal Testing.

### MRI

The MRI experiments were performed using a 9.4 T/21-cm (BioSpec 94/20 USR, Bruker, Germany) horizontal scanner equipped with a high-performance gradient system and a 36-mm-diameter ^1^H quadrature transmit/receive radio-frequency (RF) volume coil.

The animals were anesthetised with 2 % isoflurane in a mixture of air and oxygen delivered via a nose cone. The anesthetic was reduced to 1.7 % when animals were placed in the magnet. Electrocardiography (ECG), respiration and temperature were monitored with the Monitoring and Gating System (SA Instruments Inc., Stony Brook, NY, USA). Body temperature was maintained at 37 °C. The animals were placed prone and maintained throughout the experiments, usually 2.5 h long. Before the MRI experiments 375 µl of saline was injected intraperitoneally to prevent dehydration. A catheter was inserted into the tail vein for contrast agent administration.

The imaging slice was positioned perpendicularly to the large blood vessels (aorta, vena cava and postal vein).

**Fig. 1:**
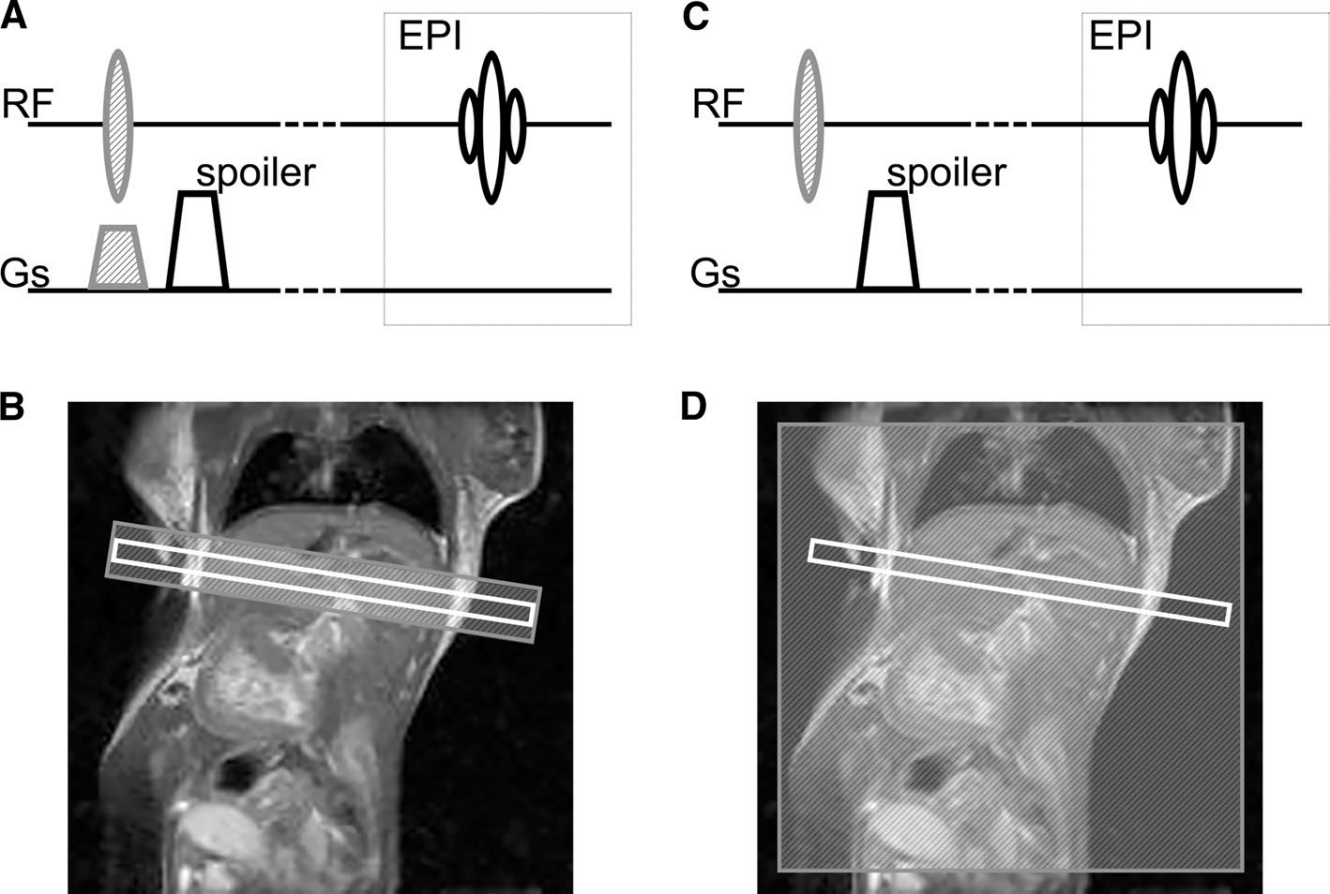
The FAIR pulse sequence with EPI readout. Two MR images are necessary for perfusion imaging, one with selective (**a**, **b**) and one with non-selective inversion of spins (**c**, **d**). **a** The RF pulse is applied simultaneously with the slice selective gradient (*shaded grey*) for the selective inversion of spins. The slice gradient is followed by the spoiler gradient for magnetisation refocussing. **b** The selective inversion is wider than the selected slice (*in white*) to ensure uniform inversion. **c** As a control image, the RF pulse is applied without the slice-selective gradient providing excitation of all spins. **d** The area of non-selective inversion covers the entire sensitive region of the RF coil. The FAIR pulse sequence is followed by the EPI sequence to create the MR image [14]

ASL imaging was conducted using the FAIR-EPI pulse sequence (Fig. 1) with the following parameters: TE = 6.324 ms, TR = 8000 ms, NA = 4, FOV = 45 × 30 mm, matrix size = 96 × 64, slice thickness = 1 mm, selective inversion slice thickness = 2.5 mm and 10 inversion recovery times (IRs) from 50 to 4500 ms. Fat suppression was used with a frequency selective 90° pulse. The total measurement time was approximately 10 min.

DCE-MRI was performed using the IntraGateFLASH™ sequence with the following parameters: TE = 0.838 ms, TR = 4.212 ms, NA = 1500 or 200, flip angle = 18°, FOV = 30 × 30 mm, matrix size = 128 × 128, slice thickness = 1 mm and navigator slice thickness = 8 mm. The total measurement time was 90 min; 10 µl/1 g b.w. of 0.0025 M hepatotropic gadolinium-based contrast agent (Primovist^®^, Bayer Schering Pharma AG, Germany) [24] was injected into the tail vein 2 min after the first image acquisition. MR images were collected in three series (150 images each), each 10 min long, a total of 30 min, to observe the contrast uptake and the maximum enhancement of liver tissue with high temporal resolution; 1500 averages were used for each series. During the remaining 1 h, one image was acquired every 10 min with 200 averages.

Immediately after the MR experiments the animals were killed, blood was collected for analysis, and the livers were harvested for histological staining.

### Data analysis

Perfusion and *T*_1_ maps were created using ASL Perfusion Macro software (ParaVision 5.1, Bruker, Germany). The *T*_1_ values were obtained from the control *T*_1_ maps with non-selective spin inversion. The threshold was applied to the perfusion maps to reject false values (higher than 500 ml/min/100 g or negative). ImageJ software (NIH, USA) was used for the thresholding. Three regions of interest (ROI) placed in the liver parenchyma were used for analysis.

The time courses of the DCE-MRI images were processed with ImageJ. An oval-shaped ROI comprising 137 pixels for each MR image was placed in the right liver lobe. The mean values (±SD) of the signal intensities within ROIs were calculated for each image. The data were processed with Origin Pro 8.6.0 (OriginLab, MA, USA). The signal-time curves were normalised by averaging the signal values of the pre-contrast images and subtracting the average value from the entire time course. The final curves were fitted with the empirical mathematical model (EMM) proposed by Fan et al. [25]:

$$ C\left( t \right) = A \cdot \left( {1 - e^{ - \alpha t} } \right)^{q} \cdot e^{ - \beta t} \cdot \frac{{1 + e^{ - \gamma t} }}{2}, $$

where *A* is the upper limit of the signal, *α* is the rate of the contrast uptake (1/min), *β* is the rate of the contrast wash-out (1/min), *γ* is the initial rate of the contrast wash-out (1/min), and *q* is the parameter related to the slope of the early uptake and the curvature of the transition from uptake to wash-out. Four additional parameters were also calculated: time to peak [*T*_peak_, (min)], enhancement slope [ES, (1/min)], area under the curve (AUC) and elimination half-life time [*T*_1/2_, (min)]. T_peak_ was calculated according to the formula proposed by Fan et al. [25]. ES was calculated as the ratio of *C*_max_/*T*_peak_, where *C*_max_ was determined directly from the signal-time graph. AUC was computed from time 0–90 min. The *T*_1/2_ was assessed by fitting the exponential curve to the wash-out slope.

### Biochemical and histopathological analysis

Following the MR measurements blood samples were obtained from the renal vein on ethylenediaminetetraacetic acid (EDTA) and centrifuged for 10 min at 1000 g at a temperature of 4 °C (Sigma 2-16PK, Sigma, Germany). Blood plasma was frozen at −80 °C and stored. The content of ALT and AST in blood plasma was determined with calorimetric methods using an ABX Penta 400 biochemical analyzer (Horiba Medical, Japan).

Following the blood sampling, the livers were harvested and fixed in 4 % formaldehyde phosphate buffer solution. The liver lobes were embedded in paraffin. The sections were 5 µm thick and were cut in two areas (two slices per area) approximately 100 µm apart. The regions with the necrotic (haemorrhagic and coagulative necrosis) and the collective tissue changes were evaluated as a percentage of the total section area. The infiltration of vessel walls by immune cells was classified on a four-point scale, where the score 0 was assigned to the tissue without infiltration of vessel walls and the score 3 was assigned to the samples in which the infiltration was noticed in each vessel. The samples were stained with haematoxylin and eosin (H&E) and Gomori trichrome (TRICHROME).

### Statistical analysis

All parametric parameters were presented as (mean ± STD); histological parameters were presented as median. The data, except for the histological information, was verified for normality with the Shapiro-Wilk test (*p* = 0.05). Parameters with normal distribution were analysed with the *t* test (*p* = 0.05). The Mann-Whitney *U* test was applied (*p* = 0.05) to qualitative parameters determined on the interval scale and parametric parameters with substantial distribution. Correlations among perfusion, EMM parameters and histological outcomes were tested with the Spearman rank order correlation (*p* = 0.05). Statistical analysis was performed with Statistica 8.0 (Stat Soft, Tulsa, OK, USA).

## Results

### Development of acute liver injury after ConA injection

#### Weight of the animals

The weight of the animals was measured just before and 24 h after ConA/saline injection. The weight of the ConA-treated group was 20.9 ± 0.8 g and 19.9 ± 1.1 g before and 24 h after ConA, respectively. The weight of the control animals remained stable at 20.1 ± 1.4 g before and 24 h after the saline injection. In both groups, the change in the weight was not statistically significant (*p* > 0.05).

#### Histopathology of the liver

**Fig. 2:**
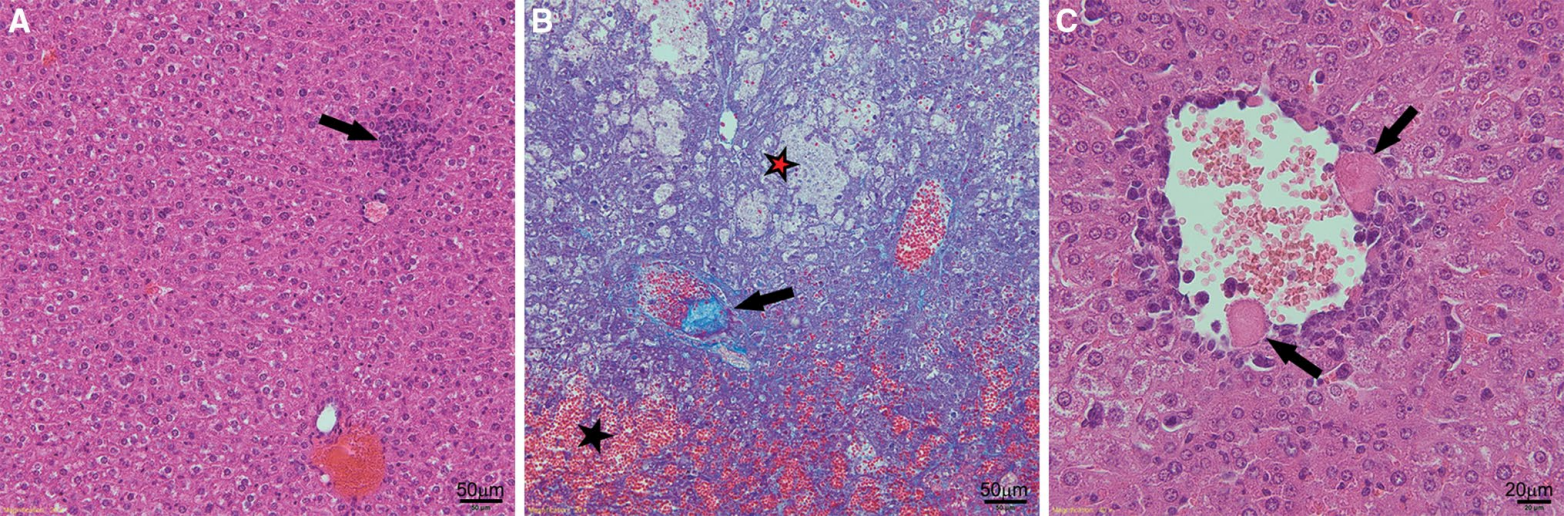
Representative microphotographs of cross sections of the livers harvested from **a** the control group (H&E staining; ×200); **b** ConA group (TRICHROME staining; ×200); **c** ConA group (H&E staining; ×400). A small inflammation focus is visible (*arrow*) in **a**. Haemorrhage (*marked with a black star*), haemorrhagic necrosis (*marked with a red star*) and embolism in a vessel (*arrow*) are visible in **b**. Hepatocyte blebs (*arrows*) are visible in **c**

Histological staining of the liver tissue from the control group showed apoptosis of single hepatocytes and traces of inflammation (Fig. 2a). No other abnormalities were observed. Histological cross-sections of the control animals were normal. In the ConA-treated group pathological changes such as hyperaemia, coagulative and haemorrhagic necrosis, inflammatory infiltration in the blood vessels wall and liver parenchyma, embolism in the vessel lumens, disintegration of the endothelium, hepatocyte blebs and haemorrhage were detected (Fig. 2b, c; Table 1). Numerous foci of necrosis scattered throughout the parenchyma in the livers of the ConA group were observed. Cellular infiltration of the parenchyma was noticed around and in the areas of necrosis (Fig. 2b).

**Table 1:** Histopathological evaluation of control mice and mice with liver injury induced by ConA

Parameter	Control group *n* = 7		ConA group *n* = 7		*p* value
	Median	Rank sum	Median	Rank sum	
Haemorrhagic necrosis [%]	0	42	0	63	0.2
Coagulative necrosis [%]	*0*	*31.5*	*10*	*73.5*	*0.009*
Vessel wall infiltration [a.u.]	*0*	*28*	*2*	*77*	*0.002*
Tissue changes [%]	*0*	*28*	*40*	*77*	*0.002*

Hyperaemia, coagulative necrosis and vessel wall infiltration were observed in the samples from the ConA group. A median and rank sum from the Mann-Whitney *U* test are shown. Test results were assumed to be significantly different at *p* < 0.05 (italics)

#### Biochemical parameters

Biochemical analysis showed substantially elevated levels of enzymes in blood plasma in the ConA group. ALT was equal to 38 ± 7 and 1627 ± 1075 U/l in the control and ConA group, respectively, while the corresponding AST values were 61 ± 15 and 1977 ± 1285 U/l. The Mann-Whitney *U* test showed that these parameters were significantly different between the groups with the following *p* values: ALT (*p* < 0.001); AST (*p* < 0.001).

### Assessment of perfusion and *T*_1_ relaxation time with arterial spin labelling

**Fig. 3:**
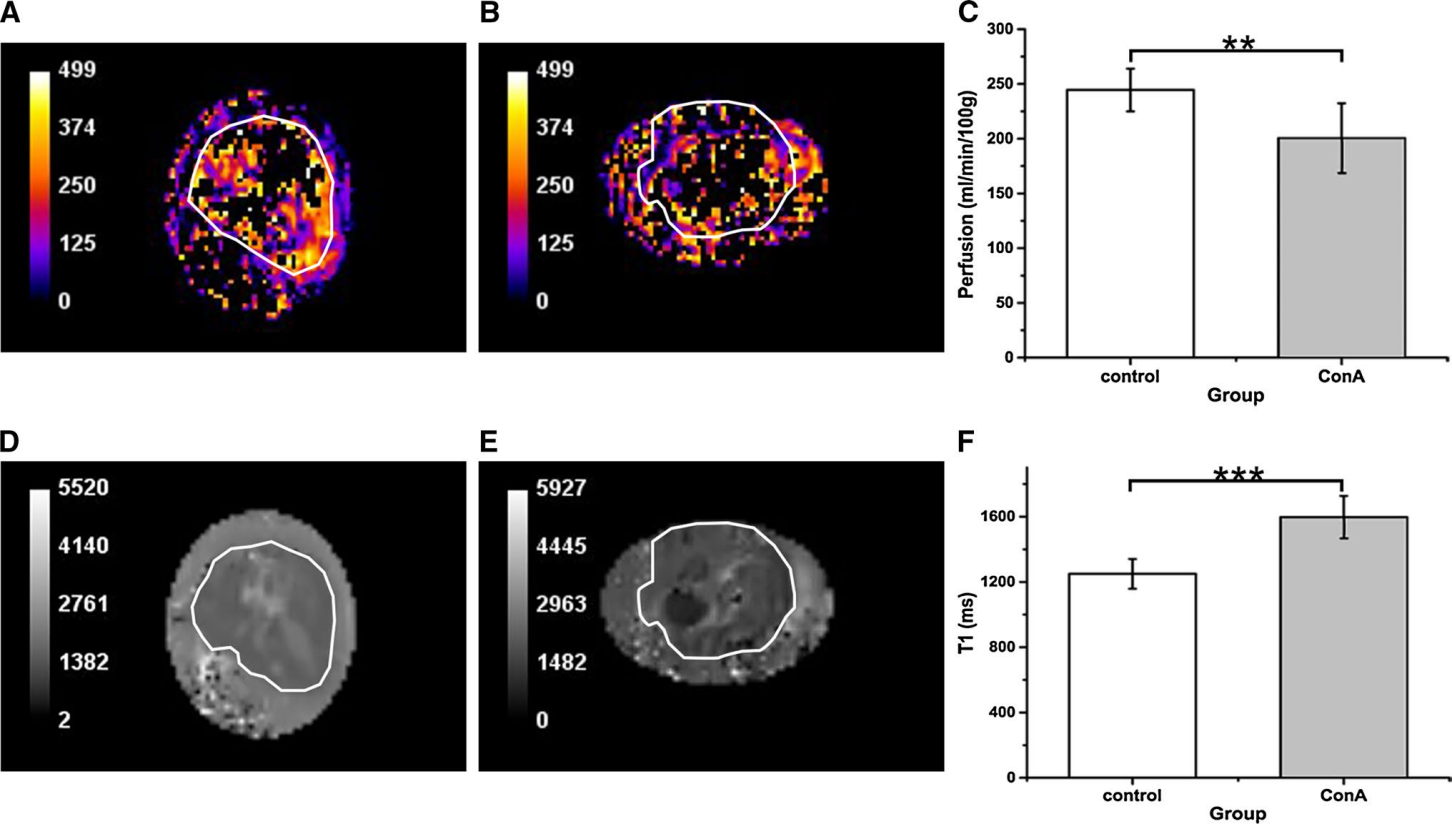
Examples of the perfusion [ml/min/100 g] (**a**, **b**) and *T*_1_ [ms] (**c**, **e**) maps. Both maps were rescaled from the original size to 256 × 171 pixels. Thresholding of pixel values greater than 500 ml/min/100 g was applied to the perfusion maps. Perfusion (**c**) and *T*_1_ values (**f**) were assessed for seven control and seven treated animals. **a** Perfusion map of the control liver. **b** Perfusion map of the ALF liver. **c** Perfusion values within ROIs in the livers of the control and ConA group. The difference in perfusion between the control and ConA group is statistically significant (**p = 0.008). **d** *T*_1_ map of control liver. **e** *T*_1_ map of ALF liver. **f** *T*_1_ values in the control and ConA group are significantly different (***p < 0.001). Areas of liver in MR images (**a**–**d**) are contoured in *white*

The examples of the perfusion maps are presented in Fig. 3a, b. Perfusion in the liver ROIs was equal to 245 ± 20 ml/min/100 g in the healthy group and 200 ± 32 ml/min/100 g in the ConA group (Fig. 3c). The *t* test indicated that the perfusion values were significantly different (*p* = 0.008) between the control and ConA group and decreased by 18 % in the treated group. A threshold was applied to the pixels with perfusion values higher than 500 ml/min/100 g of tissue. As a result, the pixels in the central area of the initial MR images were removed because of the presence of thevena cava and portal vein.

The examples of the *T*_1_ maps are presented in Fig. 3d and e. The *T*_1_ relaxation time in the livers of the control group across all animals was equal to 1249 ± 91 ms while in the ConA group it was 1597 ± 130 ms (Fig. 3f). The difference in *T*_1_ between the control and treated group was significant (*p* < 0.001).

### Assessment of the hepatocyte integrity with the DCE-MRI and empirical mathematical model

**Fig. 4:**
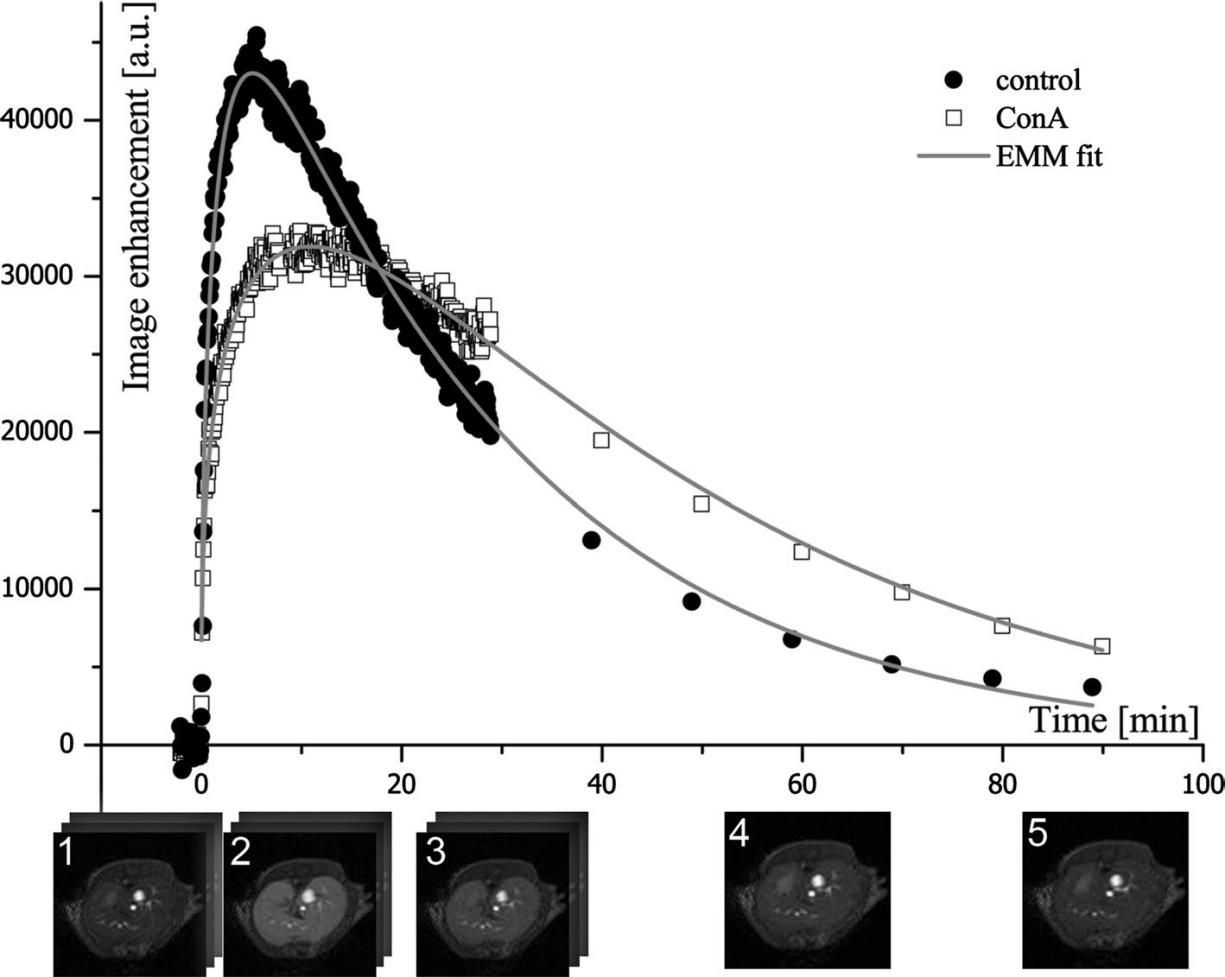
Examples of the time courses of signal changes in the control and ConA group with the fitted empirical mathematical model. The differences in the curvatures of the enhancement peaks and wash-out regions of the curves for the ConA and the control are visible. The corresponding DCE-MR images of the liver from the control group are shown beneath the horizontal axis: (*1*) pre-contrast; (*2*) 6 min after contrast injection (maximum enhancement of liver parenchyma is visible, *T*_peak_ in control group was equal to 6.14 min); (*3*) 30 min; (*4*) 60 min; (*5*) 90 min after contrast injection (contrast is washed out from the liver tissue). During the initial 30 min, MR images were acquired in three series, 150 images each. After 30 min, one image was acquired every 10 min

The Gd-EOB-DTPA compound is a hepatocyte-specific contrast agent that is taken up by living hepatocytes. As shown in Fig. 4 the Gd-EOB-DTPA signal displays different patterns in the control and ConA mice. Gd-EOB-DTPA was washed away faster in the livers of the control animals than in the livers of the ConA animals. The MR signal intensity of parenchyma in the controls returned to the pre-injection value within 90 min. In the ConA group the intensity of the liver MR images remained elevated until the end of the measurements (Fig. 4).

To further quantify differences in the Gd-EOB-DTPA wash-out between the control and ConA group, the EMM equation was fitted to the experimental data (*R* ^2^ > 0.98). The values of the parameters calculated from the EMM and *p* values are shown in Table 2. The rate of the initial wash out (*γ*) shows substantial distribution and was tested with the Mann-Whitney *U* test. The remaining parameters were tested with the *t* test. Five parameters were statistically different between the control and the ConA group: *β* (*p* = 0.004), *T*_peak_ (*p* < 0.001), ES (*p* = 0.006), *T*_1/2_ (*p* < 0.001) and AUC (*p* = 0.003). The parameters *β* and *T*_1/2_ describe the wash-out slope of the DCE-MRI curve. The significant difference in these parameters between the groups confirmed that transport of the contrast agent via hepatocytes was disturbed by the ALF processes. The value of *β* was equal to 0.037 ± 0.008 and 0.021 ± 0.008 1/min in the control and ConA group, respectively, while the corresponding *T*_1/2_ values were 31.7 ± 7.3 and 66.5 ± 10.5 min. A significant difference was also observed in the AUC parameter, which was larger in the ConA group than in the control group: 179 ± 23 × 10^4^ and 131 ± 25 × 10^4^, respectively. The uptake of the contrast agent from blood by hepatocytes was also disturbed in the ConA group as confirmed by the *T*_peak_ and ES parameters. The maximum enhancement of image intensity was noticed at 6.14 ± 1.07 min in the control group, while in the ConA group it occurred later at 9.72 ± 1.69 min. The ES value of 6587 ± 1731 and 4002 ± 1113 1/min in the control and the ConA group, respectively, confirmed that perfusion in the ConA animals was affected by ALF.

**Table 2:** Parameters obtained by fitting the EMM equation to the experimental data for both groups

Parameter	Control group *n* = 7	ConA group *n* = 7	*p* value
*A*	57,100 ± 9669	67,355 ± 31747	0.43
*α* [1/min]	0.20 ± 0.11	0.11 ± 0.08	0.1
*q*	0.432 ± 0.064	0.380 ± 0.064	0.15
*β* [1/min]	*0.037* *±* *0.008*	*0.021* *±* *0.008*	*0.004*
*γ* [1/min]	0.001 ± 0.002 (r.s.43)	0.004 ± 0.007 (r.s.62)	0.25
*T*_peak_ [min]	*6.14* *±* *1.07*	*9.72* *±* *1.69*	*<0.001*
ES [1/min]	*6587* *±* *1731*	*4002* *±* *1113*	*0.006*
*T*_1/2_ [min]	*31.7* *±* *7.3*	*66.5* *±* *10.5*	*<0.001*
AUC	*1,312,380* *±* *252,093*	*1,786,030* *±* *229,748*	*0.003*

The parameters, except *γ*, were tested with the *t* test. *γ* was tested with the Mann-Whitney *U* test; rank sum is placed in parentheses. Statistical significance was assumed at *p* < 0.05 (italics)

### Correlations between MRI-based parameters and histopathological and biochemical outcomes

The Spearman rank order correlation showed that perfusion measured with ASL correlates with the parameters calculated with EMM: *α* (*r*_S_ = 0.635), *γ* (*r*_S_ = −0.591), *T*_peak_ (*r*_S_ = −0.732) and *T*_1/2_ (*r*_S_ = −0.609). Perfusion also correlates with *T*_1_ (*r*_S_ = −0.534) and two histopathological outcomes: vessel wall infiltration (*r*_S_ = −0.766) and tissue changes (*r*_S_ = −0.565). The *T*_1_ relaxation time correlated with perfusion (in the text), *T*_peak_ (*r*_S_ = 0.754), *β* (*r*_S_ = −0.640), *T*_1/2_ (*r*_S_ = 0.859), ES (*r*_S_ = −0.582) and AUC (*r*_S_ = 0.613), coagulative necrosis (*r*_S_ = 0.689), vessel wall infiltration (*r*_S_ = 0.785), tissue changes (*r*_S_ = 0.801), ALT (*r*_S_ = 0.716) and AST (*r*_S_ = 0.736). Correlations between EMM and histological as well as biochemical parameters were also found (Table 3). All histological parameters were positively correlated with the biochemical markers (*p* < 0.05).

**Table 3:** Spearman rank order correlations between EMM parameters and histological and biochemical parameters

	*T*_peak_ [min]	*A*	*α* [1/min]	*β* [1/min]	*γ* [1/min]	*q*	*T*_1/2_ [min]	ES	AUC
Haemorrhagic necrosis [%]	*0.588*	0.279	*−0.558*	−0.224	0.282	−0.398	0.429	*−0.579*	0.322
Coagulative necrosis [%]	*0.880*	0.059	−0.522	*−0.665*	0.215	−0.445	*0.748*	*−0.782*	*0.635*
Vessel wall infiltration [a.u.]	*0.789*	0.111	−0.410	*−0.723*	0.401	−0.429	*0.756*	−0.510	*0.841*
Tissue changes [%]	*0.907*	0.000	−0.482	*−0.707*	0.229	−0.509	*0.853*	*−0.810*	*0.721*
ALT [U/l]	*0.828*	−0.159	−0.441	*−0.700*	0.216	*−0.654*	*0.784*	*−0.733*	*0.815*
AST [U/l]	*0.776*	−0.095	−0.332	*−0.723*	0.090	*−0.574*	*0.789*	*−0.631*	*0.859*

Correlations were calculated for seven animals in the control group and seven in the ALF group. Correlations marked in italics are assumed significant at *p* < 0.05

## Discussion

The aim of this study was to measure perfusion and hepatocyte injury in a mouse model of ALF induced with ConA using two MRI-based methods. The results demonstrated that the DCE-MRI and ASL techniques allow reliable assessment of alterations in liver perfusion and hepatocyte integrity induced by ConA in vivo.

ConA-induced liver injury is an established model of T-cell-mediated liver inflammation that to some extent mirrors autoimmune or viral hepatitis in humans [10]. The activated lymphocytes cause the release of pro-inflammatory mediators (e.g. INFγ, TNFα, IL-4), disruption of the endothelium and hepatocyte damage [10, 26, 27]. Our study, performed 24 h after ConA administration, allowed observing coagulative and haemorrhagic necrosis, inflammatory infiltration in the blood vessel walls and liver parenchyma as well as elevated levels of biochemical markers. All changes resembled the lesions in hepatitis, confirming the previous reports on this model [6, 28].

The ASL study showed that perfusion in the liver parenchyma in the control animals was 245 ± 20 ml/min/100 g, which is in good agreement with the previously reported values of 220 ± 30 ml/min/100 g in Balb/c mice obtained using the FAIR-ASL technique with the Look-Locker readout [29]. In our study, perfusion in ALF was impaired by 18 %, likely due to the inflow of activated and therefore inflexible and more viscous leucocytes into the site of inflammation [30–32]. The sinusoids may also be plugged by the active platelets, which circulate in blood and are not cleared by the damaged liver [8, 9]. However, some authors indicated that ALF was associated with inappropriate vasodilation and vasoconstriction [7, 9].

The *T*_1_ relaxation time was higher in the ConA group than in the control group. The *T*_1_ value in the control group was 1249 ± 91 ms, which is comparable to the previously reported 1360 ± 60 ms [29]. The correlation between *T*_1_ and perfusion suggested that the change observed in the value of the relaxation time is linked with the haemodynamics. The increased *T*_1_ time in the ConA group may be caused by water accumulation in the diseased tissue. Blood flow through the liver may be inefficient because of vessel embolisms with platelets and sequestrate leucocytes as well the reduction of the sinusoid size caused by the disturbed vasoconstrictors and vasodilator secretion [9].

Although liver function assessed by the DCE technique is also determined by liver perfusion, hepatic uptake of the Gd-EOB-DTPA contrast agent is strongly dependent on the hepatocyte integrity as Gd-EOB-DTPA is selectively taken up by the hepatocytes via the organ-anion transporters (OATPB1/B3) and excreted via ATP-dependent multidrug resistance proteins (MRP2/3/4) [24, 33, 34]. The high values of the R^2^ coefficients confirmed a very good fit of the EMM equation [25] to the DCE-MRI data. No differences between the animal groups in the rate of contrast uptake (*α*), initial rate of contrast uptake (*q*) and maximal amplitude of image enhancement (*A*) were observed. Interestingly, α correlated with the perfusion value obtained using ASL. Furthermore a difference was observed in the *T*_peak_ and ES parameters, which may indicate alterations in the blood circulation, liver filtering function and hepatocyte integrity. The *α*, *T*_peak_ and ES parameters were previously assigned as perfusion indicators [19, 20], which was confirmed in our study by the obtained correlations. At the same time we also observed correlations of these parameters with pathological changes in histological staining, supporting the theory that contrast uptake and signal enhancement depend on several factors. The wash-out slope of the DCE-MRI curve in the ConA animals was affected by disturbed transport of the contrast agent via hepatocytes because of inflammation and the recovery processes. The differences between the groups were visible in the shape of the wash-out slope and the intensities of the liver images obtained during the final stage of MR experiments. The deteriorated liver function was apparent in the elimination half-life time (*T*_1/2_) value and the rate of the contrast wash-out (*β*). Both of these parameters indicated significantly prolonged contrast removal in the ConA group. Their good correlation with histology (coagulative necrosis, tissue changes and vessel wall infiltrations) as well as blood markers implies an association with damaged hepatocytes. Although the differences observed in the histological samples between the groups are larger when compared to the differences obtained in the MRI parameters, it should be stressed that MRI provides in vivo measurements corresponding to pathophysiological processes and enables noninvasively assessing the progression or regression of the liver disease.

The drawback of the ASL technique is its low in-plane spatial resolution (0.469 mm/pixel). The low resolution of the obtained images and need to remove the pixels with extreme values introduced additional complications to the data processing and analysis. The 500 ml/min/100 g threshold was applied to exclude the blood vessels and the pixels with negative values from the MR images. Additionally, the circulatory bed in the liver has a complex structure containing not only arteries and veins but also the portal triads and sinusoid. Hence it is difficult to extract only the parenchymal area in the image.

DCE-MRI data were collected using the self-triggered IntraGateFLASH™ sequence. In this method cardiac and respiration cycles are derived from the MR signal and are used to reduce artifacts without sacrificing temporal resolution, which is especially important during the first passage of the bolus, providing information on perfusion [35, 36]. The data in this study were collected at high temporal resolution (less than 4 s per image) during the contrast wash-in phase.

Clinical evaluation of patients with ALF is based on the King’s College Hospital criteria, which incorporate the aetiology of the disease and blood tests [37]. The imaging modalities used in the diagnosis of ALF include X-ray imaging, Doppler ultrasound, electroencephalography (ECG) and echocardiograms [37]. Nevertheless, MR has been used in clinical studies of hepatitis, mostly for the assessment of liver fibrosis and cirrhosis in chronic liver failure using MR elastography [38, 39], diffusion-weighted MRI [40, 41] and DCE-MRI [41–43].

The American College of Radiologists [44] indicated that MRI, with or without a contrast agent, is a suitable technique for clinical imaging of several liver diseases. The application of ASL to measure liver perfusion in humans has been also reported with promising results [45, 46]. Hence the results of our research, supported by the previous studies, suggest possible applications of our approach to clinics, in particular for assessing ALF physiology and patient monitoring, either during onset of the liver disease or, for example, after liver transplantation.

## Conclusion

We have demonstrated that retrospectively gated dynamic contrast-enhanced MRI (DCE-MRI) with the hepatocyte-specific contrast agent and arterial spin labelling (ASL) technique allows monitoring of the alterations in hepatocyte integrity and in perfusion of the microcirculation in murine hepatitis induced by ConA. The results correlated with the histopathological and biochemical parameters of liver inflammation and injury. The results suggest that both the self-triggered IntraGateFLASH™ and FAIR-EPI sequences are well suited for comprehensive imaging of liver injury in mice at 9.4 T. This method could be used to investigate the effects of pharmacological treatment on the progression or regression of liver disease in other models of liver injury. The IntraGateFLASH™ and FAIR-EPI pulse sequences could be implemented in the clinical settings to potentially improve the ALF diagnosis in patients by simultaneous assessment of alterations in liver perfusion and hepatocyte integrity in hepatitis or other liver injuries. Hence the method may prove useful in the diagnosis and therapy of liver diseases.
